# MiR-320b aberrant expression enhances the radioresistance of human glioma via upregulated expression of ALDH1A3

**DOI:** 10.18632/aging.204617

**Published:** 2023-03-29

**Authors:** Ping Mao, Tuo Wang, Ke Gao, Yi Li, Changwang Du, Maode Wang

**Affiliations:** 1 Department of Neurosurgery, The First Affiliated Hospital of Xi'an Jiaotong University, Xi'an, Shaanxi 710061, China; 2 Department of Radiotherapy, The First Affiliated Hospital of Xi'an Jiaotong University, Xi'an, Shaanxi 710061, China

**Keywords:** radioresistance, miRNA-320b, ALDH1A3, human glioma, miRNAs

## Abstract

Accumulating evidence has demonstrated that ALDH1A3 is closely associated with development, progression, radioresistance and prognosis in a variety of cancers. However, the upstream miRNA that plays in the ALDH1A3 signaling pathways in regulating the radioresistance of glioma remains unclear. In this study, ALDH1A3 was enriched in high-grade glioma and was determined to be essential for radioresistance in GBM cell lines. Moreover, miR-320b was identified as an upstream miRNA that interacts with ALDH1A3. Low expression of miR-320b was associated with poor prognosis and radioresistance in glioma. In addition, overexpression of miR-320b counteracted the effects of ALDH1A3 on GBM cell proliferation, apoptosis and radioresistance when exposed to X-ray irradiation. Collectively, miR-320b may serve as a novel therapeutic target for glioma patients.

## INTRODUCTION

Glioma is the most common primary brain tumor in adults, leading to considerable mortality and morbidity [1]. Radiotherapy is the most important fundamental treatment for glioma patients once the maximum amount of tumor has been removed. However, the emergence of radioresistance causes tumor recurrence and has a major impact on the eventual poor prognosis of glioma patients [2]. Accumulating evidence has indicated that aberrant miRNA expression is one of the major causes of tumor radioresistance [3–5]. Therefore, it is urgent to identify the crucial miRNA that contributes to radioresistance and its underlying mechanism which may be helpful for the identification of a novel therapeutic target for glioma.

MiRNAs are small endogenous and non-coding nucleotides that regulate gene expression after transcription [6]. It has been reported that miRNAs play a major role in the regulation of radioresistance in multiple types of cancers [7–9]. Recent studies have shown that miR-7-5p regulates radioresistance via ROS generation that leads to ferroptosis in HeLa and SAS cell lines [10]. Overexpression of miR-195-3p enhances the radiosensitivity of nasopharyngeal carcinoma cells by targeting and regulating cyclin-dependent kinase 1 [11]. In addition, recent research has demonstrated that miR-410 plays a significant role in increasing radioresistance by regulating the PTEN/PI3K/mTOR axis [12]. However, the physiological function and radioresistance role of miRNAs in glioma remain under explored.

The ALDH superfamily is a group of enzymes that require either NADP1 or NAD as a cofactor and is the main aldehyde metabolic enzyme system in human cells, and it is composed of 19 members [13]. Additionally, ALDH1A3, a key member of the ALDH family, is closely associated with development, progression, radioresistance and prognosis in a variety of cancers including adenocarcinoma, poorly differentiated breast cancer and high-grade glioma [13]. A recent study showed that the inhibition of ALDH1A3 hinders the metastasis of human colorectal cancer via the miR-200-ZEB1/SANI2 axis [14]. Furthermore, it has been revealed that miR-187 drastically reduces ALDH1A3 expression and promotes the proliferation of prostate cancer cells [15]. Nevertheless, the role that miRNAs play in the ALDH1A3 signaling pathways in regulating the radioresistance of glioma remains unclear.

In this study, ALDH1A3 was found to be essential for radioresistance in GBM cell lines. Further, miR-320b was identified as an upstream miRNA that interacts with ALDH1A3. Moreover, low expression of miR-320b was associated with poor prognosis and radioresistance in glioma. Overexpression of miR-320b counteracted the effects of ALDH1A3 on GBM cell proliferation, apoptosis and radioresistance when exposed to X-ray irradiation for varying lengths of time and doses. Altogether, miR-320b may serve as a novel therapeutic target for glioma patients.

## MATERIALS AND METHODS

### Cell culture

All GBM cell lines (U251, A172, U87, LN229) and human cortical neuronal cells (HCN2) were obtained from The Cell Bank of Type Culture Collection of the Chinese Academy of Sciences. These cells were cultured in DMEM (HyClone; Cytiva) supplemented with 10% fetal bovine serum (FBS, HyClone; Cytiva) and 1% penicillin-streptomycin (Thermo Fisher Scientific, Inc.). All the cells were maintained in a humidity cell incubator at 37°C with 5% CO_2_.

### Clinical samples

A total of 106 pairs of glioma tissue and adjacent normal tissue were obtained from glioma patients who were undergoing brain surgery at The First Affiliated Hospital of Xi’an Jiaotong University between April and October 2021. The usage of glioma and adjacent normal tissue involved in this study were approved by the Ethics Committee of the First Affiliated Hospital of Xi’an Jiaotong University (Xi’an, Shaanxi, China 710061; Approval. 2020-G13). All the necessary consent forms were signed for using clinical patient samples.

### Cell transfection

The miR-320b mimic (5′-AAAAGCUGGGUUGAGAGGGCAA-3′) and negative control (NC mimic) were purchased from RIBOBIO (Guangzhou, China). Meanwhile, pcDNA3.1 and pcDNA3.1-ALDH1A3 were purchased from Gene Pharma (Shanghai, China). U251 and A172 cells (5 × 10^4^ cells per well) were cultured in a six-well plate incubated at 37°C overnight with 70% confluence according to the manufacturer's protocol. Then, the cells were transfected with the specified plasmids using the Lipofectamine^®^ 3000 kit (Thermo Fisher Scientific, Inc.) according to the manufacturer's instructions. The cells were collected for further experimentation 48 hours after transfection and the transfection efficiency was evaluated by qRT-qPCR and Western blotting.

### RNA isolation and quantitative PCR

The TRIzol^®^ reagent (AccuRef Scientific) was utilized to isolate RNA in accordance with the manufacturer's instructions. The RNA concentration was measured with the Nanodrip 2000 (Thermo Fisher Scientific, Inc.) and cDNA was synthesized using PrimeScript^®^ RT Master Mix Perfect Real-Time Reagent kit (Takara Bio, Inc.). Additionally, qRT-PCR was performed with cDNA and SYBR Green Reagent (Takara Biotechnology Co., Ltd.) on an ABI 7500 Real-Time PCR instrument (Applied Biosystems) as the following conditions: 95°C for 5 min, followed by 40 cycles of 95°C for 15 s, 58°C for 20 s and 72°C for 10 s. GAPDH (mRNA) or U6 (miRNA) served as the internal control. Relative quantification was calculated as 2^-ΔCt^. ΔCt values = target gene mean Ct value - control gene mean Ct value. The primers involved in this study were shown as following: ALDH1A3 forward, TGAATGGCACGAATCCAAGAG and reverse, CACGTCGGGCTTATCTCCT; miR-320b forward, GCGAAAAAGCTGGGTTGAGA and reverse, AGTGCAGGGTCCGAGGTATT; GAPDH forward, GGAGCGAGATCCCTCCAAAAT and reverse GGCTGTTGTCATACTTCTCATGG; U6 forward, CTCGCTTCGGCAGCACA and reverse AACGCTTCACGAATTTGCGT.

### Western blotting

Protease inhibitor cocktail (Sigma Aldrich) was added to sample lysates for Western blotting analysis. Equal amounts of protein lysates were then loaded onto 12% precast SDS-PAGE gels and transferred to a PVDF membrane (Millipore Sigma). After a 1 h incubation with 5% skimmed milk, the membrane was incubated overnight at 4°C in a cold room with the target antibodies (1:1000). Next, membranes were incubated with HRP-conjugated secondary antibody (1:10000) at room temperature for 1 h. Protein bands were visualized using the Enhanced Chemiluminescence kit (Millipore, Sigma) and qualified using ImageJ software (version 1.49, National Institutes of Health). The following antibodies were used in the present study: anti-ALDH1A3 primary antibody (Abcam, ab129815, rabbit); β-actin antibody (Abcam, ab8227, rabbit); IgG antibody (Abcam, ab288151, mouse; negative control); anti rabbit secondary antibody (Abcam, ab288151).

### Irradiation assay

U251 and A172 cells were seeded in 6-well plates at a concentration of 5 × 10^6^ cells per well and incubated with DMEM/F12 at 37°C for 48 h. Subsequently, U251 and A172 cells were exposed to X-ray irradiation for varying lengths of time (0 h, 6 h, 12 h, and 24 h) and doses (0 Gy, 2 Gy, 4 Gy and 6 Gy).

### Cell Counting Kit-8 (CCK-8) assay

The viability of U251 and A172 cells from different groups was assessed by using the CCK-8 kit (Dojindo, Japan). Initially, 2000 cells/well were placed in 96 well plates. Then, 10 μl of the CCK-8 reagent was added to each well and incubated for 2 h at 37°C. The absorbance at 450 nm of each well was measured with an ELX800 microplate reader (BioTek Instruments, Inc.) to evaluate the cell proliferation.

### Colony formation

U251 and A172 cells were seeded into 6-well plates at a density of 500 cells per well and cultured for 14 days. Each well was performed by triplicate. The cell culture medium was every two days. Then, the cell colonies were fixed with 100% methanol at 4°C for 30 min and stained with 1% crystal violet in 20% methanol at room temperature for 30 min and the cell clones were observed under an inverted microscope.

### Flow cytometry

The Annexin V-FITC Apoptosis Detection kit was purchased from Nanjing KeyGen Biotech Co., Ltd. Apoptosis analysis was performed according to the manufacturers’ protocols. The percentage of cells in the early and late stages of apoptosis was used to calculate the amount of apoptosis.

### Dual-luciferase reporter assay

PGL3 and PGL3-ALDH1A3 Luciferase reporter gene vectors containing the ALDH1A3 promoter were purchased from Genepharm (Shanghai). The reporter gene plasmid was then co-transfected with phRL-TK into U251 cells cultured to a confluence of 70–80% in 24-well plates. Following this, 100 ng of pGL3-WT, 20 ng of the transfection control Renilla vector (pRL-TK; Promega Corporation) and 100 nM miR-320b mimic or NC mimic (Guangzhou RiboBio Co., Ltd.) were transfected into U251 cells using the Lipofectamine^®^ 3000 kit (Thermo Fisher Scientific, Inc.) according to the manufacturer’s protocol. The Bright-Glo^™^ Luciferase Assay system (Promega Corporation in Madison, WI, USA) was utilized to measure the luciferase activity of each sample, with Renilla luciferase activity being used to normalize the results.

### Bioinformatics analysis

The gene expression data and overall survival rate of ALDH1A3 and miR-320b in different WHO grades of glioma were extracted and analyzed based on the Chinese Glioma Genome Atlas (CGGA) database (http://www.cgga.org.cn/). ENCOR1, miRWalk and TargetScan database were used for selection of target miRNA for ALDH1A3.

### Statistical analysis

The statistical analysis was conducted with Prism 7.0 software (GraphPad, La Jolla, CA, USA). The data were presented as the mean ± standard deviation with three replicates. The student’s *t*-test and one-way ANOVA were used to analyze pairs or multiple groups. The log-rank test was used for the Kaplan-Meier survival analysis. Multivariate Cox stepwise regression was chosen for further multivariate survival analysis. Statistical significance was determined at *p* < 0.05.

### Data availability

The datasets are available from the corresponding author on reasonable requests.

## RESULTS

### ALDH1A3 was highly expressed in high-grade glioma

**Figure 1:**
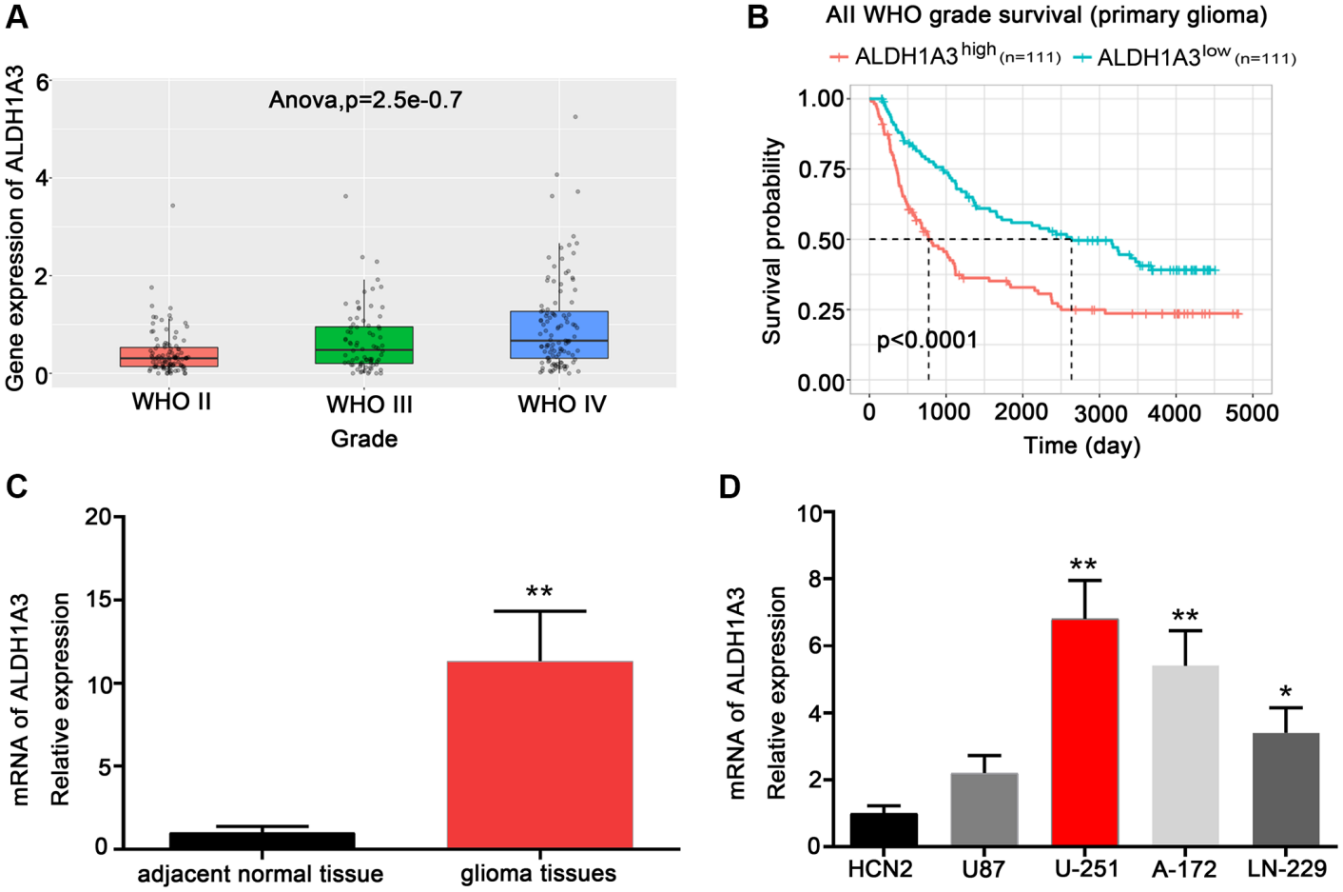
**ALDH1A3 was highly expressed in high-grade glioma.** (**A**) ALDH1A3 expression in different WHO grades of glioma based on the CGGA database. (**B**) The Kaplan-Meier analysis of the CGGA database revealed that glioma patients with increased ALDH1A3 expression had a lower overall survival rate. (**C**) ALDH1A3 was highly enriched in glioma tissue compared with adjacent normal tissue. (**D**) Elevated expression of ALDH1A3 was observed in GBM cell lines compared to human cortical neurons (HCN2). ^*^p < 0.05, ^**^p < 0.01.

We investigated the amount of ALDH1A3 present in glioma by analyzing mRNA microarray data from 325 glioma samples from the CGGA database. It appeared that ALDH1A3 was expressed at higher levels in high-grade glioma than in low-grade glioma (Figure 1A). Moreover, glioma patients with a high expression level of ALDH1A3 had a shorter lifespan than those with a lower amount of ALDH1A3 (Figure 1B). The mRNA expression level of ALDH1A3 in glioma tissue was significantly higher than in the adjacent normal tissue (Figure 1C). Furthermore, qRT-PCR was used to validate the mRNA expression of ALDH1A3 to identify the most suitable GBM cell lines for further biological experiments. The results indicated that ALDH1A3 was expressed at a higher level in GBM cell lines, particularly U251 and A172, when compared to human cortical neuronal cells (HCN2) (Figure 1D).

### ALDH1A3 was essential for radioresistance in GBM cell lines

**Figure 2:**
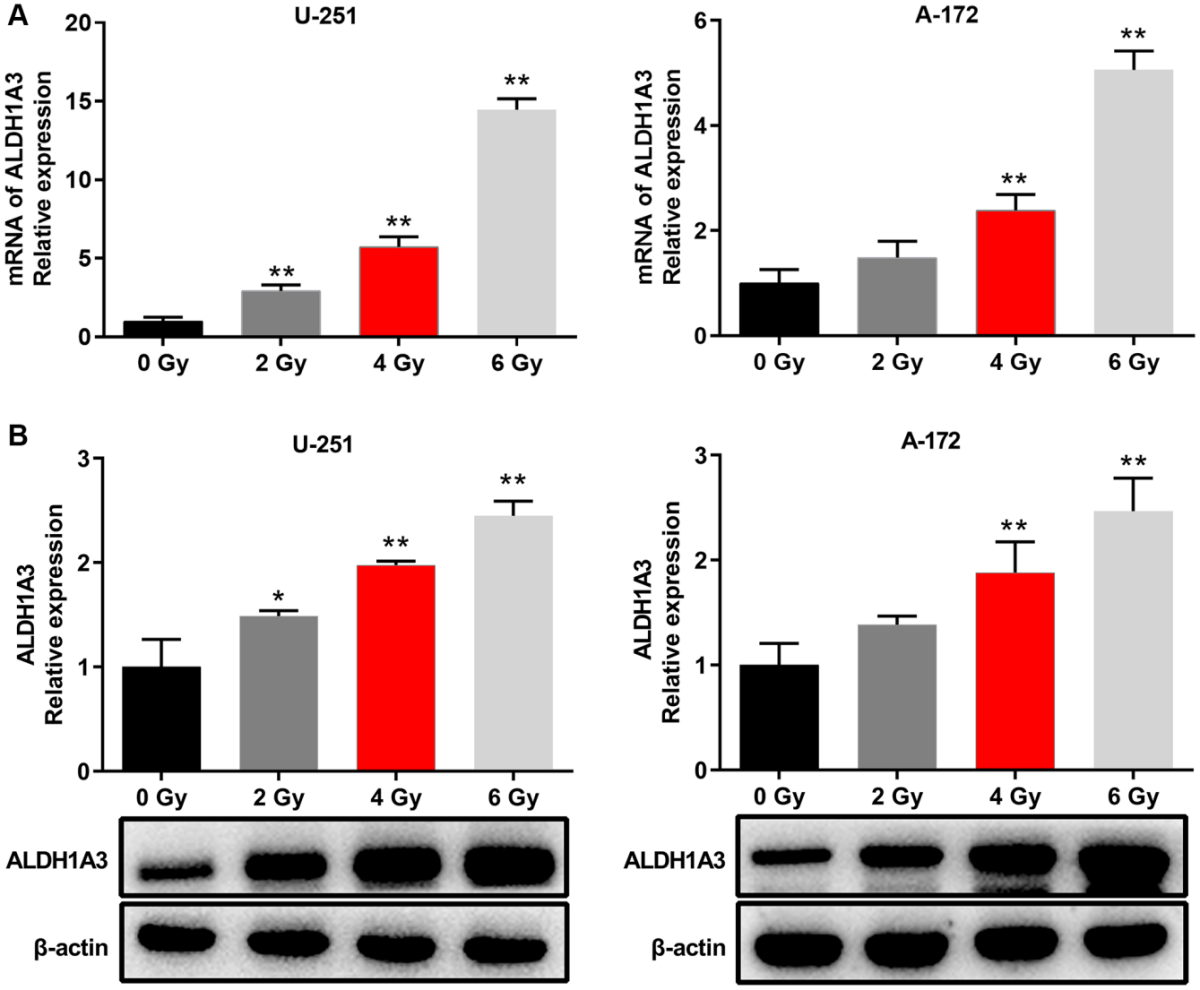
**ALDH1A3 was essential for radioresistance in GBM cell lines.** (**A**) The mRNA expression levels of ALDH1A3 when GBM cells were exposed to different doses of irradiation (0 Gy, 2 Gy, 4 Gy and 6 Gy). (**B**) The amount of ALDH1A3 protein in GBM cells after being exposed to various doses of irradiation (0 Gy, 2 Gy, 4 Gy and 6 Gy). β-actin served as control. ^*^p < 0.05, ^**^p < 0.01.

Evidence has shown that ALDH1A3 plays a key role in the chemotherapy resistance of glioblastoma by controlling autophagy during treatment [13]. However, the biological function of ALDH1A3 in radio-resistance remains under explored. We performed qRT-PCR and Western Blot experiments to explore the part ALDH1A3 plays in the radio-resistance of GBM. The results showed that both mRNA and protein levels of ALDH1A3 increased steadily in U251 and A172 cells when the irradiation dose increased from 2 Gy to 6 Gy (Figure 2A and 2B). In conclusion, these findings demonstrated that ALDH1A3 was essential for radio-resistance in GBM cell lines.

### Knockdown of ALDH1A3 enhanced radiosensitivity in GBM cell lines

**Figure 3:**
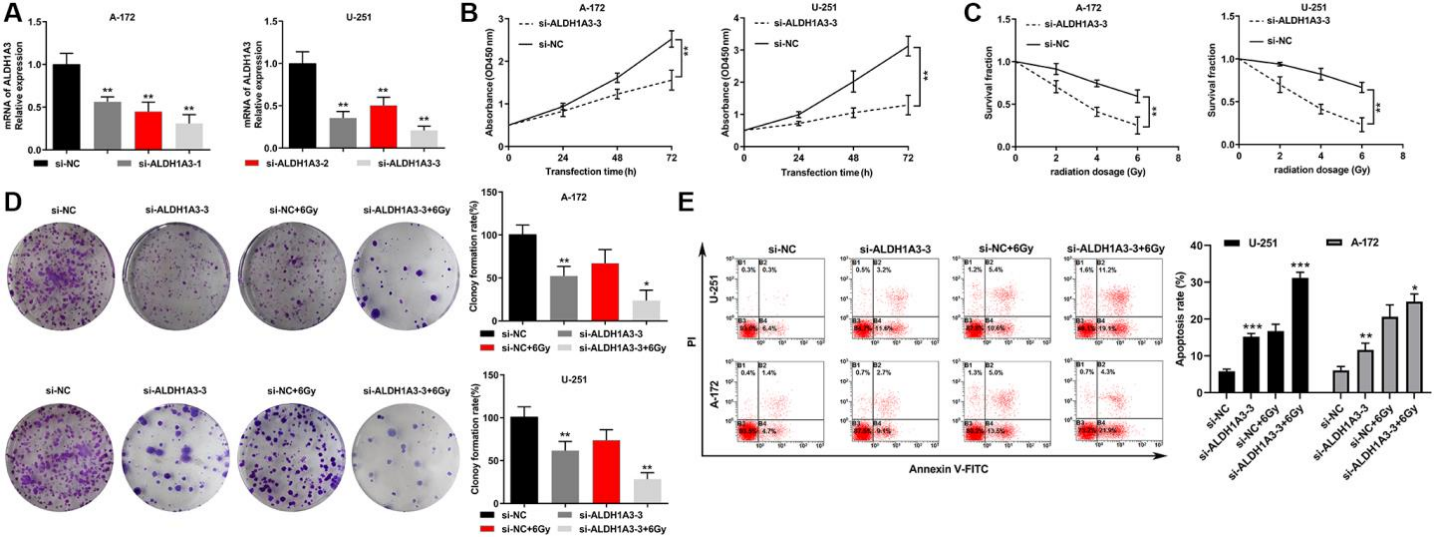
**Knockdown of ALDH1A3 enhanced radiosensitivity in GBM cell lines.** (**A**) The mRNA levels of ALDH1A3 decreased when GBM cells were transfected with siRNA. (**B**) The growth rate of GBM cells was reduced by transfection with si-ALDH1A3. (**C**) The colony formation assay showed a dramatic decrease in the survival rate of si-ALDH1A3 transfected GBM cells when exposed to different doses of irradiation (0 Gy, 2 Gy, 4 Gy and 6 Gy). (**D**) A significant decrease in cell colonies treated with si-ALDH1A3 and 6 Gy irradiation was observed compared with the control group in GBM cell lines. (**E**) The apoptosis rates of GBM cells increased significantly by silencing ALDH1A3 and exposure to 6 Gy irradiation. NC represents negative control. ^*^p < 0.05, ^**^p < 0.01.

We constructed ALDH1A3 knockdown cell lines of U251 and A172 through transient transfection to investigate the part of ALDH1A3 in reaction to various doses of irradiation (0 Gy, 2 Gy, 4 Gy and 6 Gy). Figure 3A demonstrated that si-ALDH1A3-3 was the most effective siRNA against ALDH1A3. Additionally, U251 and A172 cells that were transfected with si-ALDH1A3-3 had lower cell viability than those transfected with si-NC, as shown in Figure 3B. U251 and A172 cells transfected with si-NC had higher survival rates than those cells transfected with si-ALDH1A3-3 when exposed to irradiation (Figure 3C). The colony formation assay demonstrated a considerable decrease in U251 and A172 cell colonies that were treated with si-ALDH1A3-3 and 6 Gy irradiation in comparison to the control group (Figure 3D). Flow cytometry results revealed that the apoptosis rates of U251 and A172 cells significantly increased when ALDH1A3 was silenced, particularly for those cells exposed to 6 Gy irradiation, suggesting that the suppression of ALDH1A3 could increase the radiosensitivity of GBM cell lines (Figure 3E).

### MiR-320b is an upstream miRNA that interacts with ALDH1A3 in GBM cell lines

**Figure 4:**
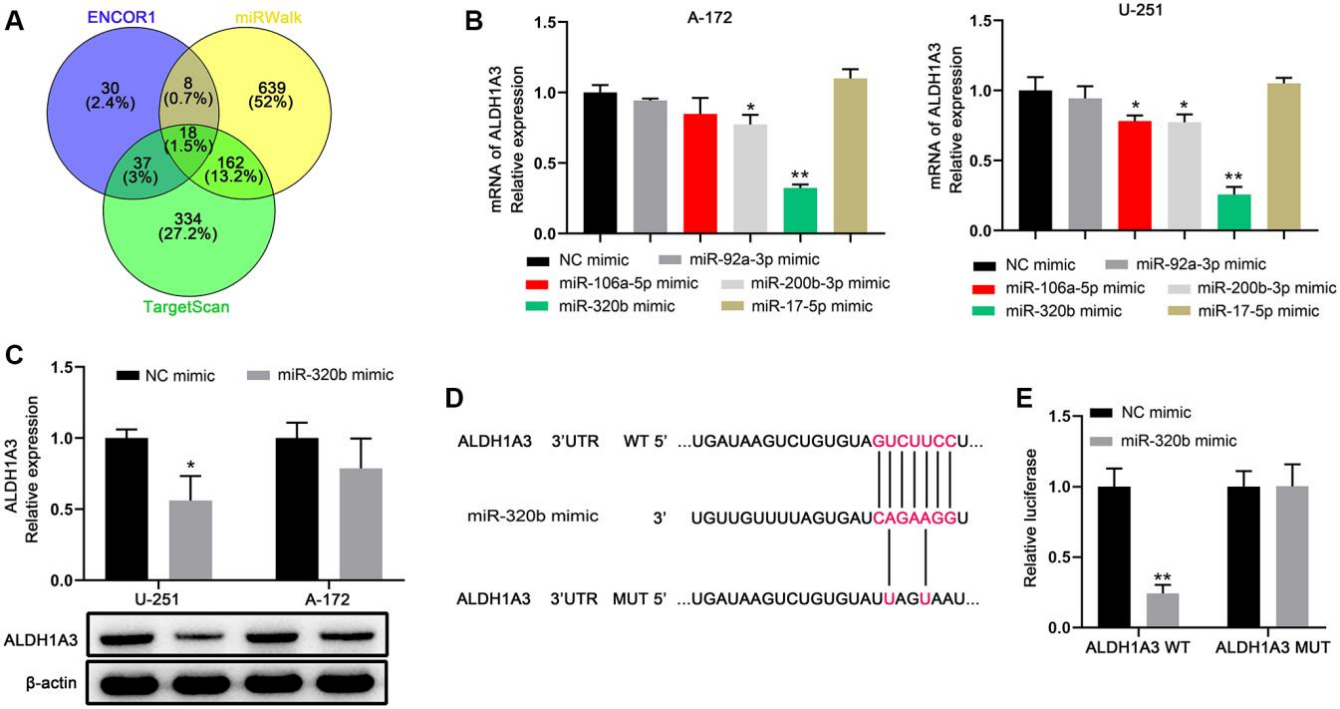
**miR-320b is an upstream miRNA that interacts with ALDH1A3 in GBM cell lines.** (**A**) The Venn diagram was used to illustrate the miRNAs that were common in the TargetScan, miRWalk, and ENCOR1 datasets. (**B**) Among the five miRNAs tested on GBM cell lines, miR-320b had the strongest impact on reducing ALDH1A3 levels. (**C**) Overexpression of miR-320b caused a decrease in the amount of ALDH1A3 protein in GBM cell lines. (**D**) The luciferase reporter constructs, ALDH1A3 3′UTR-WT and ALDH1A3 3′UTR-MUT, were created using the seed sequence of miR-320b that matched the 3′UTR of ALDH1A3 mRNA. (**E**) Luciferase activities were notably deceased after co-transfection of miR-320b mimics and ALDH1A3 WT. WT represents wild type. MUT represents mutation. NC represents negative control. ^*^p < 0.05, ^**^p < 0.01.

Bioinformatics analysis was performed to identify target miRNAs based on the consensus binding sites of ALDH1A3 in order to better understand the molecular mechanisms of ALDH1A3 promoting cell proliferation and radio-resistance in GBM cells. Ultimately, 18 predicted target miRNAs were selected through analysis of the ENCOR1, miRWalk, and TargetScan databases (Figure 4A). Additionally, miR-320b mimic had the strongest effect on decreasing ALDH1A3 expression among the five candidate miRNAs tested in GBM cell lines (Figure 4B). This was further evidenced by the fact that ALDH1A3 was reduced in U251 and A172 cells at the protein level due to the overexpression of miR-320b (Figure 4C). To verify that miR-320b could be a direct target of ALDH1A3 in GBM cells, a mutant of ALDH1A3 was constructed (Figure 4D). The results show that the luciferase activity in U251 cells was significantly reduced by the co-transfection with ALDH1A3 wild-type and miR-320b mimic compared to that of miR-320b mimic and ALDH1A3 mutant (Figure 4E). This suggests that ALDH1A3 is a direct downstream target of miR-320b in GBM cells.

### Low expression of miR-320b is associated with poor outcomes and radioresistance in glioma

**Figure 5:**
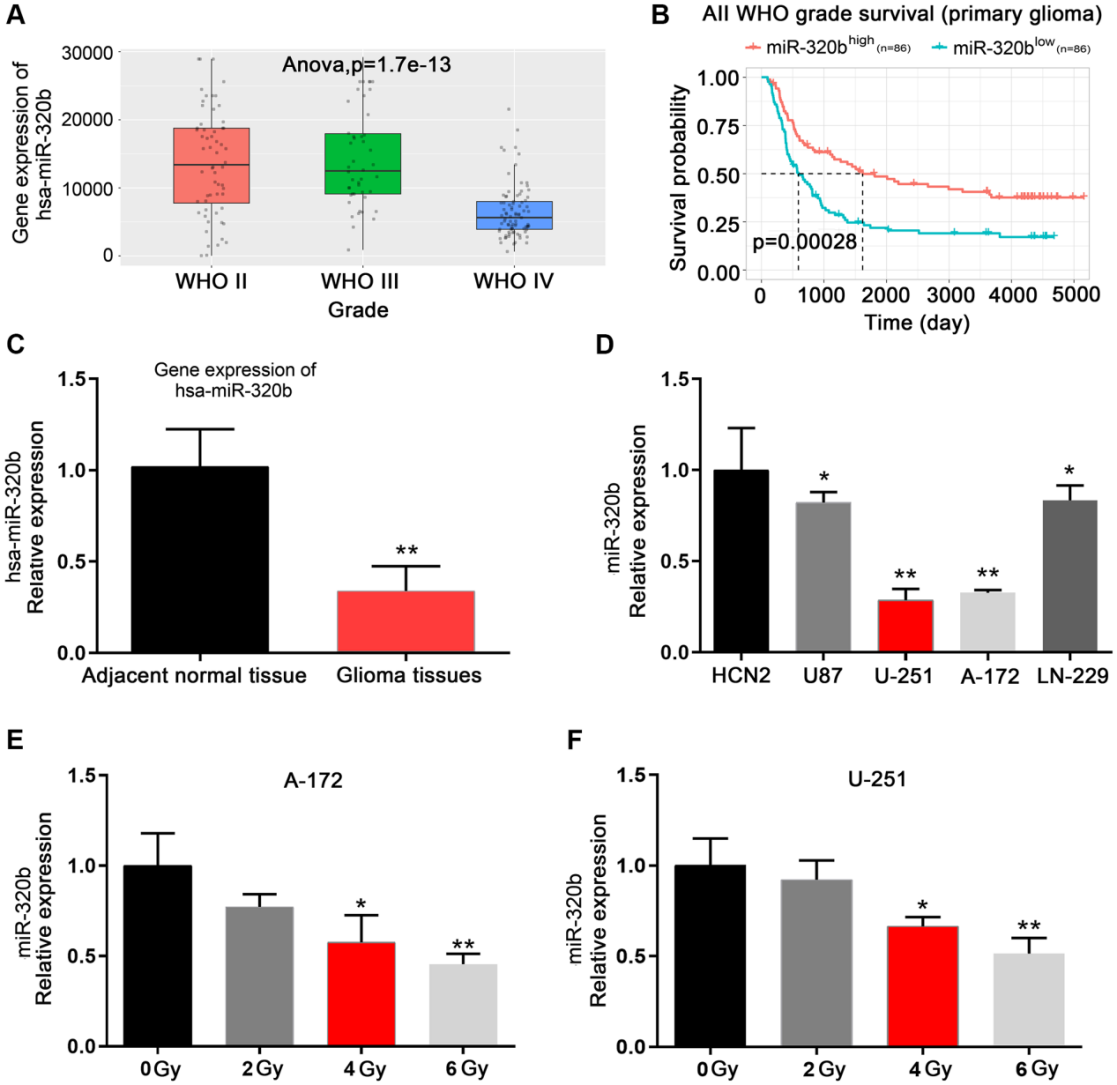
**Low expression of miR-320b is associated with poor outcomes and radioresistance in glioma.** (**A**) miR-320b in different WHO grades of glioma based on the CGGA database. (**B**) Kaplan-Meier analysis of the CGGA database showed a longer overall survival of glioma patients with elevated miR-320b levels. (**C**) miR-320b was enriched in adjacent normal tissues compared with glioma tissues. (**D**) Decreased expression of miR-320b was observed in GBM cell lines compared to human cortical neurons (HCN2). (**E** and **F**) The mRNA expression levels of miR-320b when GBM cells were exposed to different doses of irradiation (0 Gy, 2 Gy, 4 Gy and 6 Gy). ^*^p < 0.05, ^**^p < 0.01.

Analysis of the CGGA database revealed that miR-320b had a negative correlation with the WHO grade of glioma (Figure 5A). Patients with low expression of miR-320b had a worse prognosis than those with high expression (Figure 5B). Additionally, miR-320b was more abundant in normal tissues than in glioma tissues (Figure 5C). Furthermore, U251 and A172 cells had significantly lower miR-320b expression than other GBM cells. Therefore, U251 and A172 cells were selected for further irradiation assay (Figure 5D). We observed a decrease in miR-320b expression in U251 and A172 cells as the amount of irradiation exposure increased (Figure 5E and 5F). In conclusion, we determined that a decrease in miR-320b is associated with a worse prognosis and resistance to irradiation in glioma.

### Overexpression of miR-320b suppressed cell proliferation and enhanced radiosensitivity in GBM cell lines

**Figure 6:**
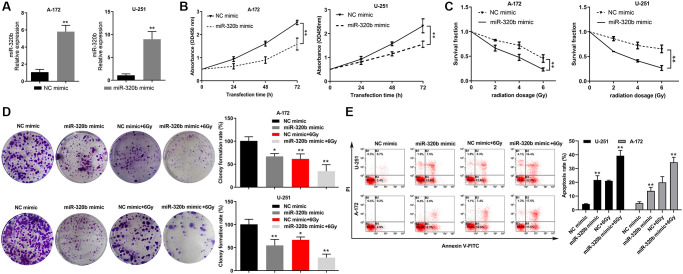
**Overexpression of miR-320b suppressed cell proliferation and enhanced radiosensitivity in GBM cell lines**. (**A**) The overexpression efficiency of miR-320b mimic was evaluated by qRT-PCR in GBM cells. (**B**) Overexpression of miR-320b attenuated cell proliferation in GBM cell lines. (**C**) The colony formation assay revealed a significant decrease in the viability of GBM cells with miR-320b overexpression when exposed to irradiation doses of 0 Gy, 2 Gy, 4 Gy and 6 Gy. (**D**) A significant decrease of cell colonies was observed in GBM cells with miR-320b overexpression, particularly when exposed to 6 Gy irradiation. (**E**) The apoptosis rates of GBM cells were increased significantly by miR-320b overexpression and exposure to 6 Gy irradiation. NC represents negative control. ^*^p < 0.05, ^**^p < 0.01.

MiR-320b was overexpressed in U251 and A172 cells to investigate its functional roles in cell proliferation and radioresistance in glioma (Figure 6A). The results showed that the overexpression of miR-320b attenuated the cell proliferation of both U251 and A172 cells (Figure 6B). Additionally, overexpression of miR-320b suppressed the cell survival rate in U251 and A172 cells when exposed to different doses of irradiation (0, 2, 4, and 6 Gy) (Figure 6C). The cell colony formation assay further revealed that overexpression of miR-320b decreased the colony-forming ability of U251 and A172 cells, especially when exposed to 6 Gy irradiation (Figure 6D). Further, the results of the apoptosis rates of U251 and A172 cells being significantly increased after the combination of miR-320b overexpression and 6 Gy irradiation suggest that miR-320b plays an important role in the radiosensitivity of GBM cells (Figure 6E). This implies that overexpression of miR-320b not only suppresses cell proliferation but also enhances the radiosensitivity of GBM cells.

## DISCUSSION

Glioma, which is the most common and devastating deadly brain tumor, has poor outcomes despite the application of various treatments including radiotherapy and chemotherapy [16]. Previous studies have revealed that radioresistance is one of the most significant factors in the low survival rate of glioma patients [17]. Accumulating evidence has indicated that aberrant expression of miRNAs is closely associated with tumorigenesis, therapeutic resistance, and tumor recurrence of a variety of cancer types [18–20]. It has been demonstrated that the aberrant expression of miR-320b is strongly involved in cancer growth, angiogenesis, metastasis and radioresistance in lung cancer, colorectal cancer, breast cancer and hepatocellular carcinoma [21–24]. Furthermore, a previous study showed that the overexpression of miR-320b could suppress cell proliferation via the Bcl-2/Bax apoptosis pathway and serve as a novel prognostic marker for glioma [25]. In this study, we identified that low expression of miR-320b was associated with poor prognosis and radioresistance in glioma. Moreover, the elevated expression level of miR-320b suppressed cell proliferation and enhanced radiosensitivity in GBM cell lines. In addition, we explored the potential mechanism of miR-320b in GBM radio-resistance.

Increasing evidence has shown that ALDH1A3 exhibits high activity in multiple cancer types and influences a diverse range of biological characteristics including cell proliferation, invasion, therapy resistance and prognosis [13, 26–28]. ALDH1A3 protein expression affects various aspects of glioma cells, including apoptosis, proliferation, cell cycle, mitochondrial membrane potential, glucose consumption, lactate production and invasion ability [29]. Furthermore, our previous study has identified ALDH1A3 as a functional biomarker for mesenchymal glioma stem cells that are resistant to radiation [30]. In this study, we found that the expression of ALDH1A3 was significant in glioma and crucial for radioresistance in GBM cell lines. Additionally, knockdown of ALDH1A3 enhanced radiosensitivity in GBM cell lines. However, the molecular mechanism of ALDH1A3 involved in radioresistance in glioma remains unclear.

A previous study revealed that the expression of miR-187 was inversely correlated with ALDH1A3 expression in prostate cancer, with miR-187 being an upstream target of ALDH1A3 [15]. Furthermore, it appears that miR-7 reduces breast cancer growth by inhibiting the activity of ALDH1A3 and diminishing the number of breast cancer stem cells [31]. To explore the potential regulatory mechanism of ALDH1A3 in glioma, we performed bioinformatics analysis of target miRNAs using the ENCOR1, miRWalk, and TargetScan databases to examine the consensus binding sites of ALDH1A3. In this study, we identified miR-320b as an upstream miRNA that interacts with ALDH1A3 in GBM cell lines. Furthermore, overexpression of miR-320b counteracted the effects of ALDH1A3 on GBM cell proliferation, apoptosis and radioresistance when exposed to different doses of irradiation.

In conclusion, our data first illustrated that aberrant expression of miR-320b enhanced cell proliferation and radioresistance via upregulated expression of ALDH1A3 in glioma. Furthermore, miR-320b may serve as a novel therapeutic target for glioma patients.
